# Predictors of clinical outcomes in space-occupying cerebellar infarction undergoing suboccipital decompressive craniectomy

**DOI:** 10.3389/fneur.2023.1165258

**Published:** 2023-04-17

**Authors:** Kristin Lucia, Sarah Reitz, Elke Hattingen, Helmuth Steinmetz, Volker Seifert, Marcus Czabanka

**Affiliations:** ^1^Department of Neurosurgery, University Hospital Frankfurt, Frankfurt, Germany; ^2^Department of Neurology, University Hospital Frankfurt, Frankfurt, Germany; ^3^Department of Neuroradiology, University Hospital, Frankfurt, Germany

**Keywords:** cerebellar, suboccipital decompressive craniectomy, GCS, outcome, predictors

## Abstract

**Introduction:**

Despite current clinical guidelines recommending suboccipital decompressive craniectomy (SDC) in cerebellar infarction when patients present with neurological deterioration, the precise definition of neurological deterioration remains unclear and accurate timing of SDC can be challenging. The current study aimed at characterizing whether clinical outcomes can be predicted by the GCS score immediately prior to SDC and whether higher GCS scores are associated with better clinical outcomes.

**Methods:**

In a single-center, retrospective analysis of 51 patients treated with SDC for space-occupying cerebellar infarction, clinical and imaging data were evaluated at the time points of symptom onset, hospital admission, and preoperatively. Clinical outcomes were measured by the mRS. Preoperative GCS scores were stratified into three groups (GCS, 3–8, 9–11, and 12–15). Univariate and multivariate Cox regression analyses were performed using clinical and radiological parameters as predictors of clinical outcomes.

**Results:**

In cox regression analysis GCS scores of 12–15 at surgery were significant predictors of positive clinical outcomes (mRS, 1–2). For GCS scores of 3–8 and 9–11, no significant increase in proportional hazard ratios was observed. Negative clinical outcomes (mRS, 3–6) were associated with infarct volume above 6.0 cm^3^, tonsillar herniation, brainstem compression, and a preoperative GCS score of 3–8 [HR, 2.386 (CI, 1.160–4.906); *p* = 0.018].

**Conclusion:**

Our preliminary findings suggest that SDC should be considered in patients with infarct volumes above 6.0 cm^3^ and with GCS between 12 and 15, as these patients may show better long-term outcomes than those in whom surgery is delayed until a GCS score below 11.

## 1. Introduction

Space-occupying cerebellar infarctions constitute only 1–4% of all ischemic strokes (1–4); however, their reported overall mortality is 15–32% (5–7). Treatment options for patients suffering from space-occupying cerebellar infarction aim at reducing parenchymal swelling in the posterior fossa and include pharmacological therapy, ventricular drainage (EVD), and suboccipital decompressive craniectomy (SDC).

Previous studies have found that 24–40% of patients with space-occupying cerebellar infarction will undergo surgical treatment by suboccipital decompressive craniectomy (8, 9). The current AHA guidelines for the management of cerebellar infarction recommend surgical decompression in patients with neurological deterioration despite maximal medical treatment with Class I, Level B evidence (10). Studies examining the efficacy of surgical treatment vs. the best medical care in space-occupying cerebellar infarction have shown that SDC provides better clinical outcomes than conservative therapy alone in patients with neurological deterioration (9–14), whereas a precise definition of “neurological deterioration” does not exist.

Despite evidence pointing toward the benefit of surgical therapy in selected patients, clear clinical or radiological criteria based on which the decision to perform SDC can be made are lacking. Daily management of patients with space-occupying cerebellar infarction is often interdisciplinary so parameters used to evaluate “neurological deterioration” should be standardized and easily applicable by the team of clinicians involved in patient care.

To this end, the Glasgow Coma Scale (GCS) score is a well-established clinical grading system that requires an evaluation of the verbal response, eye opening, and motor response to evaluate the level of consciousness. The use of the GCS score in the management of space-occupying cerebellar lesions has shown that SDC is usually performed in patients with a GCS score between 8 and 10 (6, 8, 15, 16). Clinically, this score indicates a patient with a strongly impaired level of consciousness and (with a GCS score of 8 or below) may require protective intubation.

In this retrospective analysis of 51 adult patients undergoing SDC for space-occupying cerebellar infarction, GCS scores were stratified into three categories (GCS: 3–8, 9–11, and 12–15) to examine whether clinical outcomes can be predicted by the GCS score immediately prior to SDC and, if so, to examine whether higher GCS scores are associated with better clinical outcomes. Furthermore, clinical and radiological characteristics among patients with space-occupying cerebellar infarction were analyzed as additional predictors of clinical outcomes.

## 2. Methods

### 2.1. Study design

This study was conducted as a single-center retrospective analysis, which was approved by the local ethics committee (Nr. 2022-825) and was conducted in accordance with the Declaration of Helsinki.

We retrospectively identified all patients admitted to our hospital for space-occupying cerebellar infarction between January 2010 and June 2022. Electronic medical records were used to screen which patients underwent SDC vs. standard medical therapy, thus leaving 51 patients who were then further analyzed in detail.

Electronic medical records and archived imaging data (computed tomography) were used to gather data on patient demographics, clinical history pertaining to symptoms at onset, time of clinical admission, imaging data (stroke volume, vascular territory, unilateral/bilateral stroke, Evans Index, compression of the ambient cisterns, tonsillar herniation, and hemorrhage), lysis or thrombectomy performed prior to SDC, the last follow-up, the mRS score at the last follow-up, and GCS scores. Imaging data and GCS scores were collected for each of the following time points: at symptom onset, at hospital admission, and at the surgery.

Indications for performing SDC were considered signs of neurological deterioration and the judgment of these were at the discretion of the managing physicians. Most commonly, these included deterioration in terms of GCS score, signs of hydrocephalus, brainstem compression, dilated pupils, or anisocoria.

Surgical techniques included preoperative insertion of an external ventricular drain (EVD), craniectomy of the affected hemisphere, strokectomy, and primary dural closure.

GCS scores were categorized into three separate groups: 3–8 points, 9–11 points, and as we aimed to examine whether patients with higher GCS scores than those previously described may also profit from SDC, a third group (GCS, 12–15 points) was defined. The separation of these groups was chosen to reflect clinically distinct states of neurological deterioration and values previously used in the literature (6, 8, 15, 16).

### 2.2. Imaging analysis

All analyses were performed on non-contrast computer tomographic data with a 0.2-mm slice thickness.

Infarct volumes were estimated by measuring the largest horizontal diameter (A) and its largest perpendicular diameter (B) in an axial image. The vertical diameter (C) was determined by summing the number of slices in which the lesion is visibly multiplied by the slice thickness (0.2 mm; 0.02 cm). Infarct volume was then calculated according to the formula: Volume (cm^3^) = A × B × C/2 (17).

**Figure 1:**
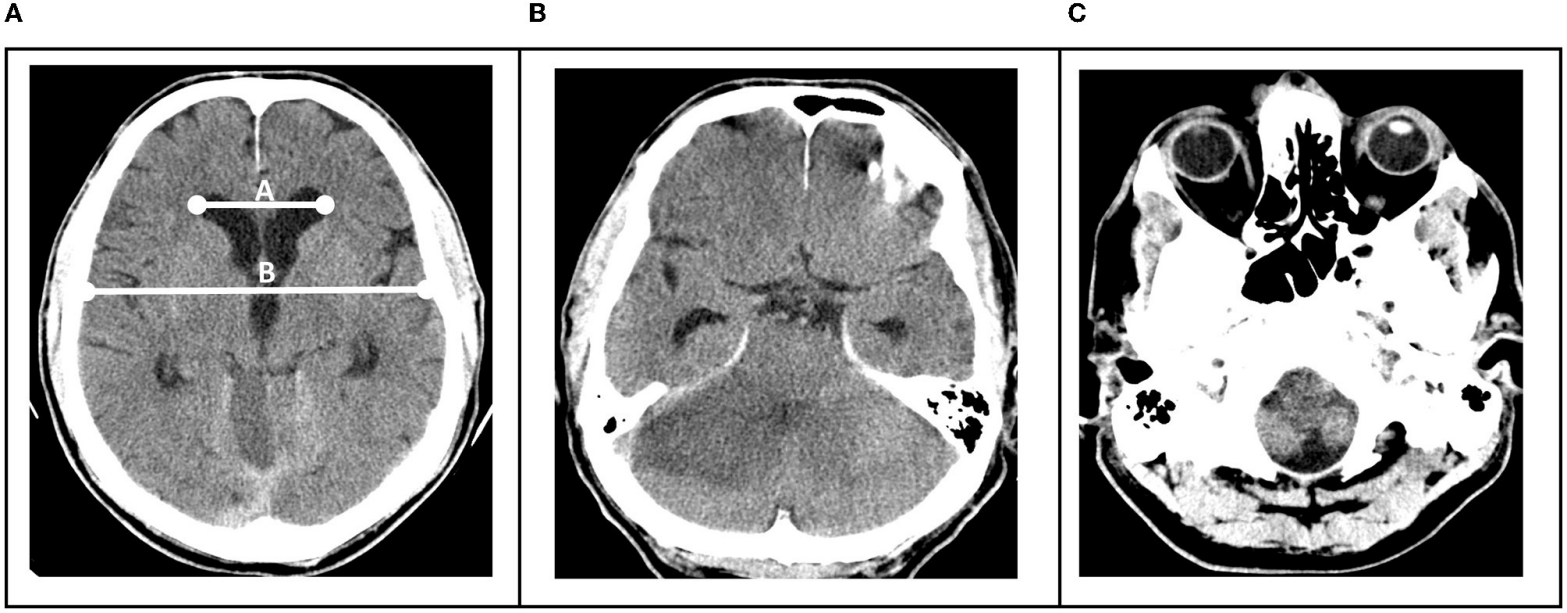
**(A)** Hydrocephalus was defined as an Evans Index of >0.3, determined by division of the distance between frontal horns of the lateral ventricles and the maximal internal diameter of the skull **(B)**. **(B)** Brainstem compression was present when uni- or bilateral occlusion of the ambient cisterns was visible. **(C)** Tonsillar herniation when tonsils were uni- or bilaterally visible below the level of the foramen magnum.

Hydrocephalus was defined as an Evans Index of >0.3, which was measured as previously described (18) (Figure 1A).

Brainstem compression was determined to be present when uni- or bilateral occlusion of the ambient cisterns was visible (Figure 1B) and tonsillar herniation when tonsils were uni- or bilaterally visible below the level of the foramen magnum (Figure 1C).

### 2.3. Statistical analysis

Quantitative values are presented as median values with a range unless otherwise noted. Group comparisons were performed using the Mann–Whitney *U*-test. Fisher's exact test was used for the comparison of categorical variables. Univariate Cox regression analysis was performed using mRS of 1–2 and mRS of 3–6 as outcome events. Multivariate Cox regression analysis was conducted using mRS of 1–2 and mRS of 3–6 as dependent variables when the univariate analysis delivered a *p*-value of < 0.015. A *p*-value of < 0.05 was considered to be statistically significant. All analyses were done with SPSS (version 24; IBM Corp.).

## 3. Results

### 3.1. Patient demographics

We analyzed a total of 51 patients who underwent suboccipital decompressive craniectomy in our institution between 2010 and 2022. The median age of patients was 62 years old (ranging from 37 to 88 years) consisting of 31 (60%) male patients and 20 (40%) female patients. The most affected vascular territory was the PICA territory (47%), followed by the vertebrobasilar territory (31%), the SCA territory (6%), and AICA and SUCA territories (2% each). In 12% of cases, more than one territory was affected. In total, 35% of patients suffered a left-sided infarct, 37% a right-sided infarct, and 28% a bilateral infarct. In 78% of patients, the etiology was determined to be thromboembolic, 14% of patients were classified as having idiopathic cerebellar infarction, and 8% of patients had vascular dissection (Table 1).

**Table 1:** Patient demographics.

**Total Patients**	**51**
Age in years (median/range)	62 (37–88)
**Gender**	
Male	31 (60%)
Female	20 (40%)
**Territory**	
PICA	24 (47%)
Vertebrobasilar	16 (31%)
Combined	6 (12%)
SCA	3 (6%)
AICA	1 (2%)
SUCA	1 (2%)
**Side**	
Left	18 (35%)
Right	19 (37%)
Bilateral	14 (28%)
**Etiology**	
Thromboembolic	40 (78%)
Idiopathic	7 (14%)
Dissection	4 (8%)
**Intervention**	
Endovascular thrombectomy	6 (12%)
Intravenous lysis	9 (17%)
**Symptoms at onset**	
Dizziness	28
Headache	11
Nausea/vomiting	25
Reduced consciousness	11
Ataxia	30
Dysarthria	10
**mRS at last follow up**	
0	0 (0%)
1	2 (4%)
2	5 (10%)
3	3 (6%)
4	16 (31%)
5	21 (41%)
6	4 (8%)
**Time elapsed (men/standard deviation) in hours**	
From symptom onset to surgery	69 (120)
From admission to surgery	52 (96)
Follow up (mean/standard deviation) in days	83 (200)

The most common symptom at onset was ataxia (30 patients), followed by dizziness (28 patients), nausea and vomiting (25 patients), headache and reduced consciousness (11 patients), and dysarthria (10 patients), respectively (Table 1).

In six patients (12%), emergency thrombectomy was performed prior to surgery. A total of nine patients (17%) received intravenous lysis therapy prior to surgical treatment (Table 1).

The mean follow-up time was 87 days following surgery (standard deviation of 200 days). At the last follow-up, two patients (4%) had an mRS of 1, five patients (10%) an mRS of 2, three patients (6%) an mRS of 3, 16 patients (31%) an mRS of 4, and 21 patients (41%) an mRS of 5. A total of four patients died during treatment (mRS, 6). The mean time from symptom onset to surgery was 69 h and from hospital admission to surgery was 52 h (Table 1). The average follow-up time for all patients was 83 days following surgery.

### 3.2. Development of GCS scores and radiological characteristics between admission and surgery

We assessed the GCS scores and radiological characteristics of patients at the time of their first admission to the hospital compared to when surgery was ultimately performed. The mean time from admission to surgery was 2.3 days. At admission, 39 patients (77%) had a GCS score of 12–15, whereas immediately preceding surgery, only eight patients (16%) remained at a GCS score of 12–15 (*p* = 0.001). A total of three patients (6%) had a GCS score of 9–11 at admission which increased to 14 patients (27%) at the time of surgery (*p* = 0.001). A total of nine patients (18%) had a GCS score of 3–8 at admission, with 29 patients (57%) displaying this score at the time of surgery (*p* = 0.005) (Table 2).

**Table 2:** GCS and radiological characteristics at admission and at the surgery.

	**At admission**	**At surgery**	***P***
	***n* (% of total)**	***n* (% of total)**	
**GCS**			
3–8	9 (18%)	29 (57%)	**0.005**
9–11	3 (6%)	14 (27%)	**0.001**
12–15	39 (76%)	8 (16%)	**0.001**
**Infarct volume (cm** ^3^ **)**			
Median (range)	1.89 (0.3–7.8)	6.20 (2.2–10.8)	**0.001**
**Hydrocephalus**	12 (30%)	44 (96%)	**0.001**
Evans Index median (range)	0.30 (0.21–0.36)	0.33 (0.28–0.40)	
Tonsillar herniation	1 (2.5%)	38 (83%)	**0.001**
Brain stem compression	12 (30%)	45 (98%)	**0.001**
Hemorrhage	3 (8%)	9 (20%)	**0.001**

GCS scores and radiological characteristics in patients at first admission and at the time of surgery. The mean time between admission and surgery was 2.96 days. Statistical analysis was performed using the Wilcoxon signed-rank test for continuous variables and the chi-squared test for categorical variables. A *p*-value of < 0.05 is considered to be statistically significant (bold). Imaging at admission was missing for 11 patients and at the time of surgery in five patients. The determination of radiological characteristics was therefore performed on *n* = 40 patients at admission and *n* = 46 patients immediately before surgery.

Imaging at admission was missing for 11 patients and before surgery in five patients. Determination of radiological characteristics was therefore performed on *n* = 40 patients at admission and *n* = 46 patients immediately before surgery. Median infarct volume at admission was 1.89 cm^3^ (ranging from 0.3 to 7.8 cm^3^) which increased to 6.20 cm^3^ (ranging from 2.2 to 10.8 cm^3^) at the time of surgery (*p* = 0.001). At admission, 12 patients (30%) showed radiological signs of occlusive hydrocephalus. Among these 12 patients, three (25%) received an EVD and were monitored until neurological deterioration occurred. Occlusive hydrocephalus at the time of surgery increased to 44 patients (83%) (*p* = 0.001). Tonsillar herniation was seen in one patient (2.5%) at admission and in 38 patients (83%) at the time of surgery (*p* = 0.001). Brain stem compression was found in 12 patients (30%) at admission and in 45 patients (98%) at the time of surgery (*p* = 0.001). Clear brainstem infarction was only observed in one patient following SDC. In three patients (8%), the hemorrhagic transformation was observed at admission and was seen in nine patients (20%) at the time of surgery (*p* = 0.001) (Table 2).

### 3.3. Predictive values of preoperative GCS scores on clinical outcomes

We performed a univariate Cox regression analysis to model the relationship of GCS scores at the time of surgery with the event of a positive clinical outcome, which was considered to have an mRS of 1–2, so that the hazard ratio can be interpreted as having a likelihood of an mRS of 1–2. This analysis showed a significant increase in the proportional hazard ratio (HR) of 6.581 (CI, 1.839–36.414) and a *p*-value of 0.031 for GCS scores of 12–15 at the surgery. This group contained five of the overall seven patients with an mRS score of 1–2. For lower GCS scores of 3–8 [HR: 1.987 (CI, 0.319–12.393); *p* = 0.467] and 9–11 [HR: 0.014 (0.000–6.473); *p* = 0.173], no significant increase in proportional hazard ratios was observed (Table 3A). In a multivariate analysis including GCS score categories and radiological characteristics, only GCS scores of 12–15 at surgery were found to be statistically significant in predicting the occurrence of a clinical outcome of an mRS of 1–2 [HR 2.136 (1.017–4.485); *p* = 0.045] (Table 3B).

**Table 3:** GCS as a predictor of positive outcomes.

**Predictor**	**Hazard ratio**	**CI (95%)**	***P***
**(A)**			
**GCS**			
3–8	0.014	0.000–6.473	0.173
9–11	1.987	0.319–12.393	0.467
12–15	6.581	1.839–36.414	**0.031**
**(B)**			
**GCS**			
3–8	0	0.00–3.906	0.951
9–11	1.788	0.267–11.988	0.261
12–15	2.136	1.017–4.485	**0.045**
**Infarct volume**			0.661
(>6.0 cm^3^)	0.588	0.055–6.332	
Tonsillar herniation	1.212	0.130–11-301	0.866
Hydrocephalus	0	0.000–2.776	0.754

**(A)**. Model summary of univariate Cox regression analysis examining the predictive value of GCS scores at the time of surgery on positive clinical outcomes defined as an mRS of 1–2. **(B)**. Multivariate analysis including three GCS categories as well as tonsillar herniation and presence of hydrocephalus as predictors of positive clinical outcomes defined as an mRS of 1–2. Brainstem compression was present in 98% of patients at the time of surgery, so this factor was not included in the multivariate analysis. Bold values indicate statistical significance.

Further analysis of clinical and radiological predictors of a negative outcome (an mRS of 3–6) following SDC was analyzed using a univariate Cox regression analysis. In this study, we found that at the time of surgery, infarct volume above 6.0 cm^3^ led to a significant increase in the proportional hazard ratio of 2.473 (CI, 1.209–5.057); *p* = 0.013. Tonsillar herniation [HR: 0.279 (CI, 0.083–0.933); *p* = 0.038], brainstem compression [HR 0.304 (CI, 0.123–0.749); *p* = 0.010], and a preoperative GCS score of 3–8 [HR 2.386 (CI, 1.160–4.906); *p* = 0.018] were found to be significantly associated with negative clinical outcomes (Table 4). GCS scores of 9–11 and 12–15, patient age above the median of 63 years, radiological findings of occlusive hydrocephalus, hemorrhagic transformation prior to surgery, and time between both ictus and hospital admission until surgery showed no significant increase in the proportional hazard ratios.

**Table 4:** Clinical and radiological predictors of negative clinical outcomes.

**Predictor**	**Hazard ratio**	**CI (95%)**	***P***
**Infarct volume**			
(>6.0 cm^3^)	2.473	1.209–5.057	0.013
Hydrocephalus	0.232	0.031–1.756	0.157
Tonsillar herniation	0.279	0.083–0.933	0.038
Hemorrhage	0.652	0.255–1.595	0.348
Bilateral infarct	0.987	0.669–1.456	0.946
**Age**			
(>63)	1.054	0.270–4.106	0.94
Brain stem compression	0.304	0.123–0.749	0.01
**GCS**			
3–8	2.386	1.160–4.906	0.018
9–11	0.625	0.275–1.420	0.261
12–15	0.349	0.107–1.136	0.08
**Time from ictus to surgery**			
(>69 h)	0.768	0.411–1.434	0.408
**Time from admission to surgery**			
(>52 h)	0.805	0.495–1.311	0.384

Model summary of univariate Cox regression analysis examining the predictive value of radiological parameters as well as age and GCS scores on negative clinical outcomes defined as an mRS of 3–6. For the predictor infarct volume, age, and time from ictus/admission to surgery, values above the respective mean were used to define event occurrence, and values below the mean were considered non-occurrence.

Subgroup analysis comparing the distribution of clinical and radiological characteristics among patients with a preoperative GCS score of 12–15 and all other GCS scores revealed no significant difference from those patients with lower GCS scores (Table 5). The follow-up time between the group of patients with a GCS score of 12–15 at the time of surgery also did not significantly differ from that of patients with GCS scores of 3–8 or 9–11 points (*p* = 0.993). Among patients with GCS scores of 12–15, surgery was indicated based on the worsening of imaging findings in three patients. In the remaining five patients, the dynamics of clinical deterioration from a GCS score of 15 at admission to a GCS score of 12 or 13 combined with extensive signs of infarct volume, brainstem compression, or tonsillar herniation were the trigger for surgery.

**Table 5:** Characteristics of patients with GCS scores of 12–15 vs. GCS scores of 3–11 at the surgery.

	**GCS 12-15**	**GCS 3-11**	
Total patients	8	43	
Age in years (median/range)	53 (38–65)	62 (37–88)	0.31
**Gender**			0.923
Male	5 (63%)	26	
Female	3 (37%)	17	
**Territory**			0.819
PICA	6 (75%)	18 (42%)	
Vertebrobasilar	2 (25%)	14 (33%)	
Combined	–	6 (14%)	
SCA	–	3 (7%)	
AICA	–	1 (2%)	
SUCA	–	1 (2%)	
**Preoperative therapy**			
Lysis	1 (13%)	8 (18%)	0.303
Thrombectomy	0 (0%)	6 (14%)	
**Side**			
Left	3 (37%)	15 (35%)	0.919
Right	4 (50%)	15 (35%)	
Bilateral	1 (13%)	13 (30%)	
**Etiology**			0.519
Thrombembolic	7 (87%)	37 (77%)	
Idiopathic	1 (13%)	6 (14%)	
**Infarct volume**			0.47
(>6.0 cm^3^)	6 (75%)	22 (58%)	
Hydrocephalus	8 (100%)	36 (95%)	0.273
Tonsillar herniation	6 (75%)	32 (84%)	0.307
Hemorrhage	2 (25%)	7 (18%)	0.566
Brain stem compression	8 (100%)	37 (97%)	0.303
**Time elapsed (mean/standard deviation) in hours**			
From symptom onset to surgery	38 (38)	60 (56)	0.087
From admission to surgery	21 (26)	50 (55)	0.093
Follow-up (mean/standard deviation) in days	84 (108)	87 (216)	0.993

Clinical and radiological characteristics among patients with GCS scores of 12–15 vs. those with GCS scores of 3–11 at the time of surgery. Statistical analysis was performed using the Wilcoxon signed-rank test for continuous variables and the chi-squared test for categorical variables. Imaging at the time of surgery was missing in five patients. Determination of radiological characteristics was therefore performed on *n* = 46 patients immediately before surgery, and for other parameters, data were available for all 51 patients. A *p*-value of < 0.05 is considered to be statistically significant.

## 4. Discussion

Studies examining the efficacy of surgical treatment vs. the best medical care in space-occupying cerebellar infarction have shown that SDC provides better clinical outcomes than conservative therapy alone in patients with neurological deterioration (9–13). Despite these findings, the clinical criteria and time point at which surgery should be performed remain loosely defined and highly variable in clinical practice (9, 10, 19).

In the current study, we, therefore, sought to stratify patients into three categories of preoperative GCS scores in order to pragmatically define the term “neurological deterioration” and to examine whether SDC performed at time points at which the GCS score is comparatively higher than previous studies reporting values between 8 and 10 may improve clinical outcomes (6, 8, 15, 16).

Our findings show that patients with higher GCS scores from 12 to 15 significantly benefitted from SDC vs. patients with GCS scores of 11 or below, independent of infarct volume. It is important to note that in our cohort, we had no patients with a GCS score of 15, rather three with GCS scores of 12 and 13, respectively, and two patients with a GCS score of 14. Surgery was indicated in these cases due to increased infarct volume in control scans performed within 72 h after hospital admission. Furthermore, we observed that preoperative GCS scores between 3 and 8 are significantly associated with poor clinical outcomes, as has been previously described (9, 13). Among the eight patients with a GCS score of 12–15 prior to SDC, no further clinical or radiological factors differed significantly from the rest of the cohort indicating the potential importance of preoperative GCS scores in predicting clinical outcomes.

Whereas, our analysis shows that patients with GCS scores of 12 and higher preceding SDC show better clinical outcomes than those with GCS scores of 11 and lower, the concept of “preventative” SDC has been evaluated in a retrospective matched case–control analysis among selected patients in a single center with GCS scores of 9 or higher (16). Among patients who remained clinically stable with an initial GCS score of 9 or higher over the first 72 h following stroke ictus, it was found that SDC and the absence of brainstem infarction were independently associated with positive outcomes (mRS of 0–2) at 12 months follow-up vs. propensity-matched controls receiving the best medical treatment alone or delayed surgery upon neurological deterioration to a GCS score below 9 at time points after 72 h (16). In our cohort, no cases of brainstem infarction were observed preoperatively. One patient with a preoperative GCS score of 8 displayed brainstem infarction following SDC. This patient was one of four patients with an mRS of 6.

A GCS-based evaluation of surgical candidates was also examined by a prospective German–Austrian series of 84 adult patients with space-occupying cerebellar infarction, which found that the overall risk for poor outcomes depended on the level of consciousness after clinical deterioration (OR 2.8) (9). Although the level of consciousness was not defined in terms of GCS scores (rather “awake/drowsy, somnolent/stuporous”), a direct comparison to our results is difficult, and the central finding further highlights the importance of preoperative neurological status as a predictor of postoperative clinical outcomes.

In addition to neurological evaluation of patients with space-occupying cerebellar infarction, radiological criteria may also be considered as surrogate parameters of infarct severity and may also guide the decision process in performing SDC. The pathophysiology of neurological deterioration and fluctuating levels of consciousness secondary to space-occupying cerebellar infarction can be traced back to brainstem compression/infarction, tonsillar herniation, and hydrocephalus (20). Brainstem infarction in particular has been deemed to be a significant predictor of negative clinical outcomes (21).

In our cohort, 30% of patients showed radiological signs of brainstem compression at admission which then increased to 98% immediately preceding surgery. As brainstem compression was also observed in all patients with GCS scores of 12–15 preceding surgery, radiological criteria alone may not be sufficient to predict neurological deterioration or indicate SDC. Furthermore, MRI imaging studies of the brainstem have confirmed that brainstem compression alone cannot reliably predict neurological deterioration (22) so clinical observation with regular neurological evaluation should not be replaced by imaging alone. In addition to brainstem compression, radiological signs of tonsillar herniation and an infarct volume of >6.0 cm^3^ were positively associated with negative clinical outcomes in our cohort, with infarct volume showing the largest effect (HR = 2.47).

An additional aspect of the practical management of patients with space-occupying cerebellar infarction is the time at which critical swelling and possible neurological deterioration may occur. A commonly observed period during which radiological progression of cerebellar swelling and clinical deterioration occurs is within 72 h following ictus (7, 9, 21, 23). In our cohort, SDC was performed 69 h following symptom onset (median). In this study, we observed that the time point of surgery either before or after 69 h was not predictive of negative clinical outcomes (HR 0.769; CI 0.411–1.434, *p* = 0.408) but rather the preoperative GCS score. Although patients with GCS scores of 12–15 underwent surgery at an earlier time point than those with GCS scores of 3–11, there was no statistically significant difference between the two (21 vs. 50 h). These findings underscore the importance of clinical observation and GCS assessment when determining the need for SDC regardless of the time point at which the patient may present after symptom onset.

Although this study focuses on the role of SDC in space-occupying cerebellar infarction, the use of CSF drainage via an external ventricular drain (EVD) as an isolated treatment of occlusive hydrocephalus over SDC has been proposed (24). The results of a meta-analysis of over 700 patients with space-occupying cerebellar infarction found that treatment using EVD alone was performed in up to 18.4% of cases (13). A retrospective analysis of long-term clinical outcomes in patients treated with EVD alone vs. EVD plus SDC found that those patients treated with SDC plus EVD had higher NIHSS scores at the last follow-up compared to those treated by EVD alone (25). Furthermore, a Japanese series of 25 patients found that in patients with initial GCS scores below 9, primary SDC showed improved clinical outcomes vs. those treated first with EVD alone (11). EVD insertion combined with SDC and strokectomy, on the other hand, has been found to be associated with lower mortality in patients with space-occupying cerebellar infarction (23).

We therefore also examined the effect of occlusive hydrocephalus among patients in our cohort prior to surgical treatment in relationship to mRS at the last follow-up. Whereas, infarct volume above 6.0 cm^3^, brainstem compression, and tonsillar herniation immediately preceding surgery were significantly associated with negative clinical outcomes (mRS ≥ 3), hydrocephalus was not associated with negative outcomes. We, therefore, conclude that in patients with any of the abovementioned accompanying radiological signs, management using EVD alone is not sufficient, whereas SDC more adequately addresses brainstem compression and herniation.

Regarding surgical strategies in SDC, no clinical standard exists to date; however, previous studies including patients with different surgical treatments have revealed SDC with strokectomy to be a more commonly performed procedure vs. SDC alone (13). All patients in our cohort were treated with craniectomy, strokectomy, and intraoperative EVD placement, thereby reducing possible technical confounders due to various intraoperative treatments. Further studies are warranted to specifically address the optimal surgical procedure for SDC.

A further important aspect of clinical management in space-occupying cerebellar infarction is patients' age and whether older patients may profit from surgical intervention in relation to age-related perioperative risks needs to be identified. A Swedish series of 32 patients with unilateral space-occupying cerebellar infarction found that advanced age was not associated with poor outcomes (15), two additional studies found that advanced age was associated with poor outcomes (7) and that younger patients had better mRS and NIHSS at discharge than older patients (25). Our analysis confirmed that age above 62 years did not significantly affect the clinical outcomes following SDC, suggesting that surgical decompression can be considered a viable treatment option in older adults.

Finally, it has been proposed that space-occupying cerebellar infarction affecting certain vascular territories is associated with increased mortality. In contrast to a previous study in which infarcts in the PICA territory were found to be associated with negative clinical prognosis, our analysis found no significant predictive value of infarct territory and clinical outcomes (1). As has been previously described, the PICA territory was also the most commonly affected territory in our series (2), although we found no significant predictive role for vascular territory on clinical outcomes.

The major limitation of the current study is the monocentric, retrospective design among a small study population. Due to the comparatively low frequency of space-occupying cerebellar infarction, multicentric cooperation to further the implementation of clinical standards regarding surgical intervention is needed. Furthermore, the use of CT as a standard imaging method was chosen due to its uniform availability in clinical practice and our cohort. However, we cannot rule out that brainstem infarction, which was not detectable on CT imaging, may have been present in some patients if MRI had been performed, therefore possibly contributing to worse clinical outcomes.

## 5. Conclusion

Our preliminary results highlight the relevance of the preoperative GCS score as a simple clinical tool to determine when SDC may be performed in patients with space-occupying cerebellar infarction. Based on our findings, we propose that surgical intervention (SDC) should be considered in patients with infarct volumes above 6.0 cm^3^ with GCS scores higher than previously described in the literature as these patients may show better long-term outcomes than those in whom surgery is delayed until a GCS score of 11 or lower. Further studies are necessary to support these recommendations in clinical practice.

## Data availability statement

The raw data supporting the conclusions of this article will be made available by the authors, without undue reservation.

## Ethics statement

The studies involving human participants were reviewed and approved by University Hospital Frankfurt. Written informed consent for participation was not required for this study in accordance with the national legislation and the institutional requirements.

## Author contributions

KL and MC conceptualized the study. KL collected and analyzed patient data, wrote the manuscript including figure and table production, and performed statistical analysis. SR, EH, HS, VS, and MC reviewed and approved the final manuscript. All authors contributed to the manuscript and approved the final submitted version.
